# Isolated Marked Inferior Vena Cava Dilatation: Unusual Presentation or Underrecognized Common Phenomenon?

**DOI:** 10.1155/2018/8396523

**Published:** 2018-11-29

**Authors:** Sneha R. Gadi, Benjamin K. Ruth, Alan Johnson, Sula Mazimba, Younghoon Kwon

**Affiliations:** ^1^Department of Medicine, Emory University, Atlanta, GA, USA; ^2^Division of Cardiovascular Medicine, University of Virginia Health System, Charlottesville, VA, USA

## Abstract

Inferior vena cava (IVC) diameter and respirophasic variation are commonly used echocardiographic indices to estimate right atrial pressure. While dilatation of the IVC and reduced collapsibility have traditionally been associated with elevated right heart filling pressures, the significance of isolated IVC dilatation in the absence of raised filling pressures remains poorly understood. We present a case of an asymptomatic 28-year-old male incidentally found to have IVC dilatation, reduced inspiratory collapse, and normal right heart pressures.

## 1. Introduction

The use of echocardiogram for the assessment of inferior vena cava (IVC) size and right atrial (RA) pressure estimation dates back several decades and has undergone extensive analysis [1–5]. Current American Society of Echocardiography (ASE) recommendations [6] state that IVC diameter ≤ 2.1 cm that collapses >50% with a sniff suggests normal RA pressure (RAP, range 0–5 mmHg), whereas IVC diameter > 2.1 cm that collapses <50% suggests high RAP (range 10–20 mmHg). We report the case of an asymptomatic, previously healthy, 28-year-old male recently diagnosed with chronic hepatitis B who was incidentally found to have a dilated IVC with reduced inspiratory collapse but with normal RAP by right heart catheterization (RHC).

## 2. Case Presentation

**Figure 1:**
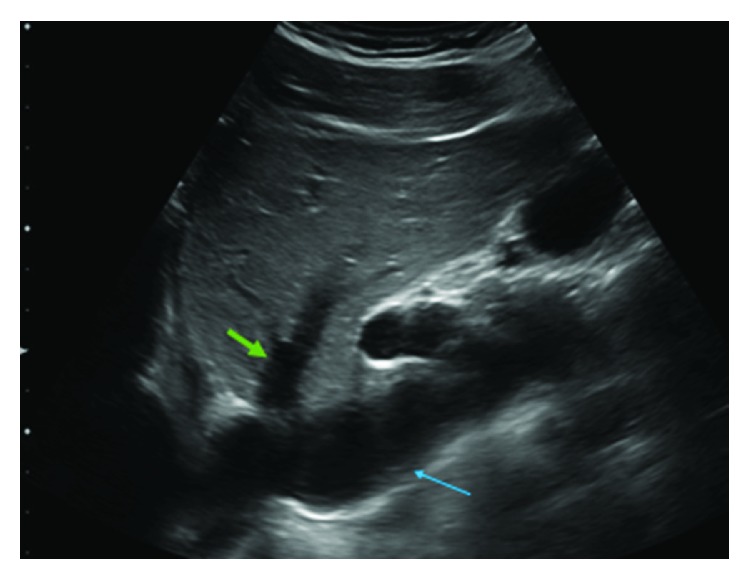
IVC on right upper quadrant ultrasound. Visualization of the hepatic vein dilatation (green) as well as the IVC dilatation (blue). Also visualized is the right atrium in the left lower corner.

**Figure 2:**
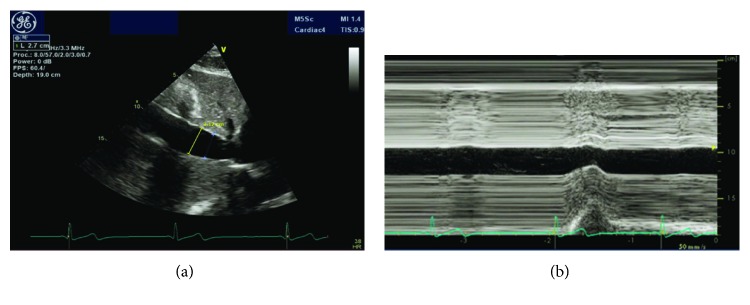
(a) IVC on transthoracic echocardiogram. Dilated IVC measuring 3.12 cm. (b) IVC on M-mode. M-mode with inspiration with maximum diameter measuring 3.12 cm and minimum diameter of 2.5 cm.

**Figure 3:**
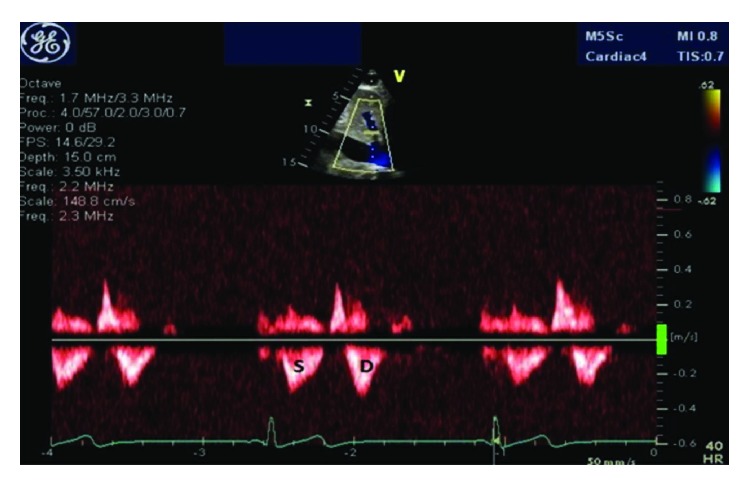
Hepatic flow Doppler. Doppler signal marked S for systolic and D for diastolic. Informal visualization shows systolic velocity (Vs) of 0.32 m/sec and diastolic velocity (Vd) of 0.38 m/sec.

A 28-year-old male was referred to our cardiology clinic for evaluation of a dilated IVC incidentally noted on liver ultrasound. The patient had recently been diagnosed with chronic hepatitis B on screening lab studies done at the local health department. The patient was asymptomatic and had no other chronic medical conditions. His social history was pertinent for multiple same-sex partners. A screening HIV test was negative. He had no family history of heart disease. As part of hepatitis work-up, the patient underwent liver ultrasound, which revealed dilated IVC and hepatic veins (Figure 1). Liver biopsy showed evidence of chronic hepatitis but no elements of cirrhosis. He was subsequently referred to cardiology for further evaluation due to concern for elevated right-sided heart pressures. Vital signs were unremarkable with blood pressure 106/62 mmHg and a pulse of 52. He had normal body habitus (body mass index of 25.8 kg/m^2^). His physical exam showed no evidence of jugular venous distention, lower extremity edema, or abdominal distention, and his cardiac exam revealed regular rate and rhythm with no murmurs or gallops. Electrocardiogram showed sinus bradycardia with normal axis, no conduction delays, and no repolarization abnormalities. Labs were pertinent for AST/ALT elevations to 200 s but normal renal function and normal brain natriuretic peptide (BNP) level. The patient subsequently underwent a transthoracic echocardiogram. This showed mildly enlarged biventricular cavity (LVIDd 6.0 cm, basal RVIDd 4.50 cm) with normal systolic function (LVEF 55–60%, TAPSE 3.10 cm, TV S′ 13.0 cm/sec, and RV fractional area change of 47%) and normal diastolic function (MV E velocity 82.56 cm/s, MV A velocity 39.53 cm/s, MV E/A 2.09, MV e′ septal 15.00 cm/s, MV e′ lateral 16.00 cm/s, E/E′ ratio 5.42, estimated PASP 34, and LA volume index 41.31 mL/m^2^). There were no significant valvular abnormalities except mild mitral and tricuspid regurgitation. RA was normal in size. The IVC diameter was increased at 2.7–3.0 cm and demonstrated <50% collapse with inspiration (Figures 2(a) and 2(b): 2D and M-mode images with markings). Dilated hepatic vein was again demonstrated, and the Doppler-based hepatic vein flow pattern showed slightly greater diastolic velocity (Vd) than systolic velocity (Vs) (Figure 3). There was no narrowing of the IVC suggestive of external compression. Given these abnormal findings suggestive of increased RAP despite an otherwise healthy young male without signs of heart failure, a RHC was performed. Hemodynamics were found to be within normal limits: mean RAP of 8 mmHg, mean pulmonary artery pressure of 19 mmHg, pulmonary capillary wedge pressure (PCWP) of 12 mmHg, and thermodilutional/Fick CI of 3.31/2.82 L/min/m^2^, respectively.

## 3. Discussion

The use of echocardiography in the noninvasive estimation of cardiac hemodynamics, particularly in relation to RAP, has undergone significant progress since first used several decades ago. Natori et al. [1] in 1979 were the first to describe the sonographic relationship of IVC size and respirophasic variation of IVC. Many studies have since shown good overall correlation between these IVC indices and RAP [2–5]. Brennan et al. [4] derived optimal cutoff values using receiver operating characteristic analysis from patients undergoing RHC and echocardiographic assessment of IVC. They found that IVC size greater than 2.0 cm and collapsibility less than 40% yielded excellent negative predictive value (90% for both) but comparatively low positive predictive value (62% for both) in predicting an elevated RAP (>10 mmHg). A different study performed by Lee et al. [7], conducted among Japanese population, reported lower optimal cutoff values (IVC size 19 mm and collapsibility 30%). Similar to the study by Brennan et al., these cutoff values yielded high negative predictive values (98% for both IVC size and collapsibility) but low positive predictive values (17% for IVC size and 20% for IVC collapsibility). Thus, one can argue that our patient's findings may simply represent a false positive echocardiographic finding of abnormal IVC index in the setting of normal RAP. However, the marked degree of dilatation of the IVC (3.1 cm) with normal RAP is rather unusual. Additionally, high RAP was suspected based on hepatic vein enlargement and abnormal hepatic vein flow pattern (i.e., systolic flow velocity (Vs)/diastolic flow velocity (Vd) < 1 (predominant diastolic)). Despite this, no evidence of high RAP was found on RHC.

There are some reported cases of IVC enlargement in the setting of normal RAP, including highly trained athletes, patients with large body-surface area, young adults with history of vasovagal syncope, those on mechanical ventilation, or those with structural causes such as narrowing of the IVC-RA junction, web or tissue present in the IVC, or prominent Eustachian valve [5, 8, 9]. Our patient did not have any of these above conditions. In one of these studies, Kim et al. [8] described a case of isolated IVC dilatation in which the authors reported “normal” RAP in the setting of abnormal echocardiographic IVC index (dilated IVC (2.4 cm) with reduced collapsibility). However, they reported the mean RAP of 13 mmHg, which is considered high. Other reports describe idiopathic dilatation of the IVC in three young patients with otherwise normal echocardiographic findings. However, RHC was not obtained in these cases. It is also important to note that although IVC indices can be indicative of elevated RAP, the strength of the association remains uncertain.

The implication of isolated dilated IVC with reduced collapsibility, especially to the degree seen in our patient, in the absence of previously established causes, is unclear. Patients included in the previously mentioned investigations, including the two largest aforementioned studies [4, 7], had much smaller IVC size distribution. For example, Brennan et al. [4] reported a mean maximum IVC diameter of 1.8 cm (SD: 0.5), and Lee et al. [7] reported a mean IVC maximum diameter of 1.6 cm (SD: 0.5), implying that those with much higher IVC diameters such as greater than 2 or 3 cm were not included in the study. As such, the positive predictive value for those with extreme IVC size is unknown and may be higher than what has been reported. While isolated abnormal IVC index may simply point to decrease in the abdominal IVC tone [9] or reduced IVC compliance in the absence of elevated RAP, confirmation by RHC may be necessary in certain clinical context. In our case, this was pursued partly due to mildly enlarged biventricular cavities of unclear etiology.

This case highlights the existing knowledge gap in our understanding of the IVC in relation to RAP. We suspect such illustrated false positive echocardiographic findings of high RAP may not be uncommon but underrecognized in real-world settings. Existing body of knowledge about IVC-RAP relationship comes solely from biased population who underwent RHC for clinical indication. To our knowledge, no data from a community cohort exist, which is understandable considering that invasive RHC correlation in a healthy population would not be typically justified.

## 4. Conclusion

We present a patient with unknown etiology of marked IVC dilation and attenuated IVC collapsibility without elevated RAP. Prevalence and prognostic implications of such finding warrant further investigations.

## Additional Points

*Learning Objective*. Echocardiographic findings of IVC dilatation with reduced collapsibility have traditionally been associated with elevated right atrial pressure. However, there exist cases in which these IVC indices are actually found in conjunction with normal right heart pressures. Given the absence of studies on normal community cohorts, it is possible that such false positive estimations of right atrial pressure may not be uncommon but simply underrecognized in real-world settings.
